# Mapping of US tobacco industry: Companies, products, histories, and market shares

**DOI:** 10.18332/tpc/196476

**Published:** 2025-01-10

**Authors:** John Gong, Marina Denicoff, Rebecca Bess, Patricia Hall, Dylan Leischow, Jose Martinez, Benjamin Weiner, Rohail Khan

**Affiliations:** 1 Office of Regulations, Center for Tobacco Products, US Food and Drug Administration, Silver Spring, United States

**Keywords:** tobacco industry, data visualization, tobacco regulation, public health

## Abstract

Data visualization can communicate information clearly and effectively through graphical means. We developed an industry landscape map to help tobacco regulatory scientists and policymakers understand a high-level overview of the US tobacco industry. This kind of mapping of the market data and deep visualization of companies and their products benefits regulatory science and public health policy in supporting potential knowledge gaps in the regulated industry.

## INTRODUCTION

Data visualization is a way to present data clearly and efficiently through graphical means, such as charts, plots, and infographics^1^. The visual display of data from different sources can deliver perspective on complex data relationships and provide data-driven insights, making it easier to understand details and associations. Good data visualization should not only communicate concepts and ideas clearly, but also stimulate viewer engagement and attention^2^. Data visualization has been utilized in many scientific fields for various purposes.

The US tobacco industry is among the world’s largest producers of tobacco products^3^. It is characterized by the presence of a few well-established large companies that manufacture most units produced, as well as many medium and small companies producing a variety of brands. These tobacco companies produce a variety of tobacco product categories, including cigarettes, cigars and cigarillos, smokeless tobacco products, oral nicotine products, electronic nicotine delivery systems (ENDS), pipe tobacco, and roll-your-own tobacco. For over a century, dynamic changes, including mergers, acquisitions, and new product launches have been occurring in the US tobacco industry^4^. These dynamic changes in the landscape of the US tobacco industry are complicated and difficult to comprehensively understand without the assistance of data visualization.

To help tobacco regulatory scientists and policymakers get a high-level overview of the US tobacco industry, we used data visualization to develop a graphic summarizing information on the structure of the US tobacco industry from various available sources. This graphic depiction provides a useful overview for tobacco product regulatory science with an ‘at-a-glance’ view of the regulated US tobacco companies and information about their products, histories, and market shares – an industry landscape.

In developing this data visualization, we first searched, collected, and analyzed information gathered from various resources, including Euromonitor International^5^, tobacco company websites, published articles on tobacco market analysis^6-8^, and other internet sources. We then developed an organizational chart of the largest US tobacco companies in terms of sale values, the product brands they manufacture, and their 2022 market shares. In the resulting data visualization, we used logos of companies and product brand images from company websites to organize and list company associations and the products each company produces. For each product category, the top five companies were ranked by the percentage of their US market shares (sales divided by revenue) in their respective product category. The graphic also provides a brief history of each company and reflects the transmission of products and brands with the mergers or separations of various tobacco companies.

## COMMENTARY

**Figure 1:**
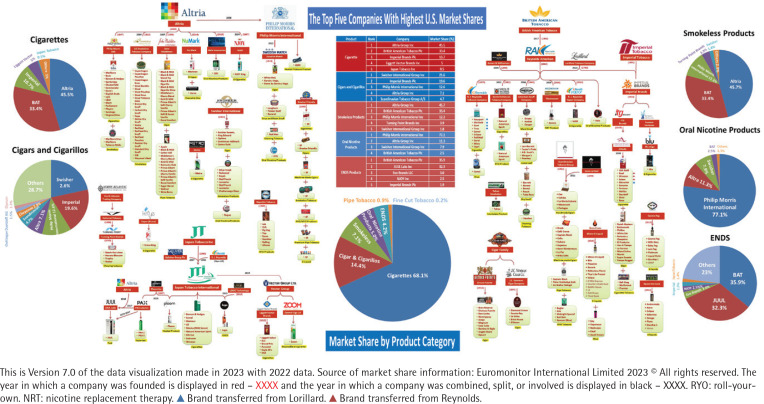
US tobacco industry overview

This data visualization was initially created in 2015 and has been updated and enriched annually with the most recently available market share data from Euromonitor and other resources data. Figure 1 shows the US tobacco industry data visualization based on 2022 data for the market shares and 2024 for other industry information. The pie chart in the center of Figure 1 shows the market share of each category of tobacco products in 2022: cigarettes at 68.1%, cigars and cigarillos at 14.4%, smokeless products at 7.1%, oral nicotine products at 5.0%, ENDS at 4.2%, pipe tobacco at 0.9%; and, fine-cut tobacco at 0.2% of the overall market for tobacco products. In 2022, oral nicotine products, as a new product category, exceeded ENDS to become the fourth-largest tobacco product category in the rank of market shares. The table above the pie chart in the center of Figure 1 lists market shares of the top five companies for each of the top five major types of tobacco products. The top five tobacco companies represent more than 76% of the market; for cigarettes, the same five companies represent 95% of the market.

The relationship between the tobacco companies and their brands is depicted via the visualization of information (company names and company brands and images) as well as data regarding market shares by company for each of the tobacco categories (pie charts). For example, Altria is currently the largest tobacco company in the US, starting in 2003 after Philip Morris Cos. was renamed. Altria’s Marlboro is the top cigarette brand listed among the top brands that Altria owns. Altria and its products are listed on the left side of the data visualization. The second tobacco giant, BAT (British American Tobacco), described on the right side of the data visualization, entered the US market by purchasing Reynolds American in 2017. Two years before the acquisition, in 2015, Reynolds bought a major part of Lorillard and released a small portion of Reynolds to Imperial. In Figure 1, those product brands are shown via different color arrows as transferred from Lorillard to Reynolds or Imperial (blue) or transferred from Reynolds to Imperial (red).

### Limitations

One of the limitations of data visualization is the ability to include a complete set of data and information. Due to the limited space, we focused on the companies whose market shares rank in the top five and represent most of the US market. Many small companies and their products are not included in this data visualization.

## CONCLUSION

The graphic attempts to provide an overview of the US tobacco industry landscape using data visualization methods. It summarizes the organization of large US tobacco companies and the types and brands of the tobacco products they manufacture and sell. The graphic is easily updated as business relationships change and products enter and exit the market. This data visualization of market information and relationships between companies and their products also provides a very useful overview to quickly find related data or information needed by tobacco regulatory scientists and policymakers. We have received positive feedback on the accuracy and functionality of the application from tobacco regulatory scientists and policymakers both within and outside the FDA.

## Data Availability

The data supporting this research are available from the authors on reasonable request.
